# Solid Pseudopapillary Neoplasms Are Rare, Indolent Pancreatic Tumors in Young Women

**DOI:** 10.1155/2020/6694904

**Published:** 2020-11-23

**Authors:** Amin Dalili, Mohsen Aliakbarian, Mehdi Karimi-Shahri, Alireza Samadi, Sara Raji

**Affiliations:** ^1^Surgical Oncology Research Center, Faculty of Medicine, Mashhad University of Medical Sciences, Mashhad, Iran; ^2^Department of Pathology, Faculty of Medicine, Mashhad University of Medical Sciences, Mashhad, Iran; ^3^Gastrointestinal and Liver Diseases Research Center, Mashhad University of Medical Sciences, Mashhad, Iran; ^4^Student Research Committee, Faculty of Medicine, Mashhad University of Medical Sciences, Mashhad, Iran

## Abstract

*Introduction*. Solid pseudopapillary neoplasm (SPN) is a rare and indolent pancreatic tumor with low malignant potential which frequently occurs in reproductive-age females. Complete resection is almost always the curative option. *Case Presentation*. We present a 20-year-old woman with acute epigastric pain and vomiting in multiple episodes. Abdominal ultrasound showed a hypoechoic lesion with the probable source in the pancreas. Following that, CT scans and Endoscopic Ultrasound (EUS) manifested a 9 × 7.5 cm-sized hypodense mass with heterogeneous well-defined margins in the pancreas suggesting the diagnosis of SPN. Whipple's procedure was performed. Histopathological examination and immunohistochemistry confirmed SPN without evidence of malignancy. *Discussion*. SPN is known as a tumor with a favorable prognosis and a long survival rate after complete resection. However, some literature focused on minimally invasive surgery as an alternative surgical approach.

## 1. Introduction

Solid pseudopapillary neoplasm is a rare tumor with low malignant potential, accounting for 1-2% of exocrine pancreatic neoplastic lesions [1]. In advance of WHO redefining it as a solid pseudopapillary tumor of the pancreas in 1996, it was named Frantz Tumor owing to Virginia Frantz, the pathologist who initially described it [2]. It mostly affects young women during their reproductive age [3–5]. Due to the remarkable improvement in clinical knowledge, imaging technologies, and immunohistochemical methods, we witness a significant progressive increase (7 times) in cases since 2000 [5–8]. SPNs are usually asymptomatic, and they are discovered incidentally while routine check-up or abdominal imaging exams are performed for other reasons [4, 7]. As SPNs have an excellent prognosis, and the complete resection is a curative treatment, accurate and prompt diagnosis by imaging modalities and pathological examinations are critical. Complete resection is a choice of treatment upon the site of the tumor [1, 2], but also, other authors argued about the priority of minimally invasive pancreatectomy may be more effective [9, 10]. In the present case, we aim to elucidate the critical aspects of this rare pancreatic tumor with accompanying splenic cyst.

## 2. Case Presentation

A 20-year-old former healthy Iranian woman referred to our tertiary clinic with acute, sharp epigastric pain and vomiting. The patient gave a history of similar pain episodes, which resulted in referring to other emergency departments and received conservative treatment. Other than this was unremarkable. She denied weight loss or diarrhea and had no jaundice. On general physical examination, there were no significant points. Her family history was negative for relevant gastrointestinal malignancies.

On admission, routine laboratory tests, including complete blood count, liver function test, electrolytes, venous blood gas, blood sugar, urine analysis, and urine culture, were within a normal range. Except for mild anemia, no other things were seen. With doubt of any gynecologic pathologies such as ectopic pregnancy, beta-human chorionic gonadotropin (BHCG) was measured, which was unremarkable.

In the emergency department, complete abdomen and pelvic ultrasound (U/S) were requested, which showed a hypoechoic lesion with 8 × 8 cm size with a probable pancreatic origin and suggested a CT scan with and without contrast to be more accurate.

**Figure 1:**
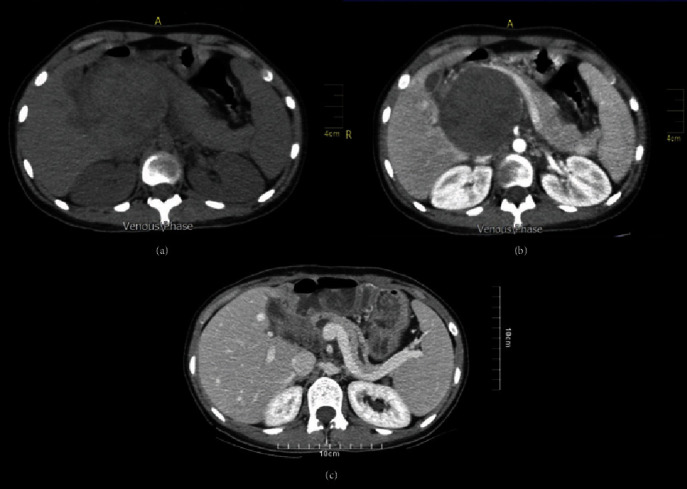
Computed tomography scans (a) without contrast and (b) with contrast before the surgery showed a hypodense mass in the epigastric area between the liver, kidney, and pancreas. (c) Postsurgical contrast-enhanced computed tomography.

Abdominal contrast-enhanced computed tomography (CECT) scan revealed a 7.5 × 7 hypodense mass in the epigastric area between the liver, kidney, and pancreas. Also, a 5 cm simple splenic cyst was reported (Figure 1). It suggested an evaluation of the hydatid cyst. Hence, hydatidAb (IgG) was requested which was negative.

**Figure 2:**
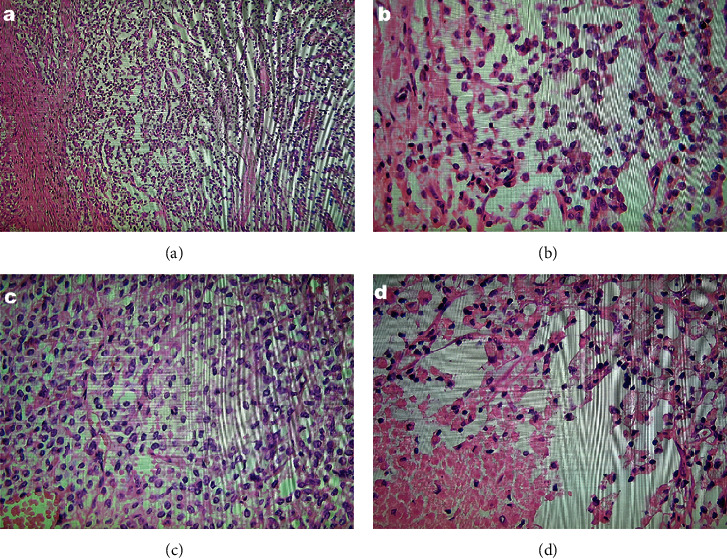
Histologic examination showed pseudopapillary (a, b) and solid components (c) of mildly pleomorphic epithelial cells with areas of necrosis and foamy histiocytes accumulation (d). Some tumoral cells contained hyaline globules within their cytoplasms (c).

**Figure 3:**
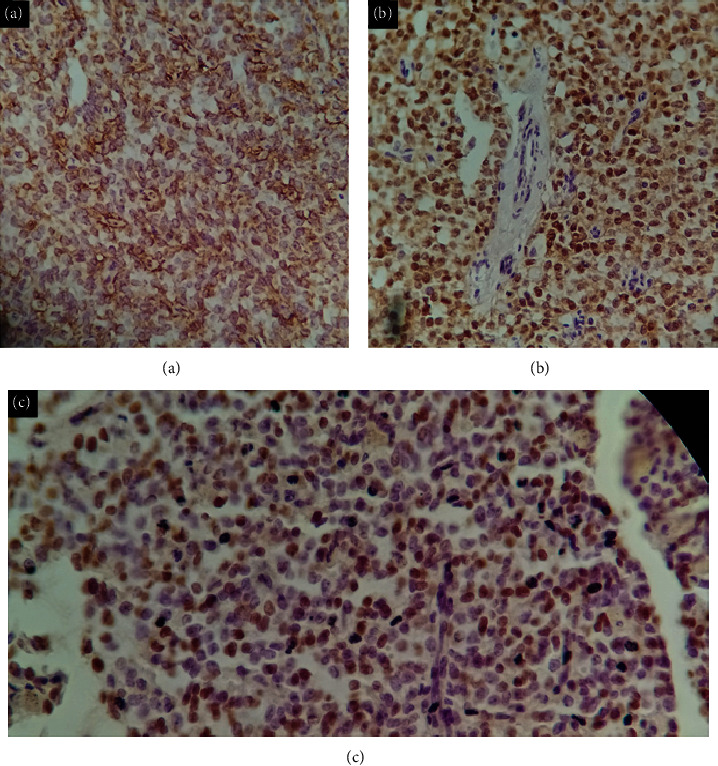
Immunohistochemistry examination: positive staining for (a) vimentin, (b) B-catenin, and (c) progesterone receptor (PR).

In an attempt to clarify the diagnosis, EUS was performed and found a 9 × 7.5 cm heterogeneous well-defined mass lesion with possible belonging to neck of pancreas (NOP) and portal vein (PV) confluence compression, so the pancreatic solid pseudopapillary neoplasm was the most likely diagnosis. A Whipple's procedure and partial splenectomy were planned. The preoperative and postoperative periods were uneventful. The patient was discharged after eight days with acetaminophen 325 mg TDS, ranitidine BID, and ferrous sulfate plus folic acid daily as well as recommended to refer to the outpatient clinic for post-op observation. The surgical specimen was sent to pathology; macroscopically, the head of the pancreas with the greatest dimension of 7 cm encapsulated with a solid, hemorrhagic, and necrotic area was described. Microscopically as shown in Figure 2, the lesion was composed of a well-circumscribed encapsulated neoplasm with solid and pseudopapillary components and large areas of necrosis. The neoplastic cells were mildly pleomorphic epithelial cells with round to oval nuclei, some with grooves and indistinctive nuclei with low mitotic activity, and pale acidophilic to clear cytoplasms. Some tumoral cells contained hyaline globules within their cytoplasms. The areas of cystic degeneration with cholesterol crystals and foamy histiocytes were observed as well. Immunohistochemical study revealed immunoreactivity for vimentin, progesterone, and beta-catenin in tumoral cells while they were negative for cytokeratin, chromogranin, synaptophysin, and CD99 (Figure 3).

The patient's postoperative recovery was smooth, and she was well without complications or signs of recurrence at five months follow-up.

## 3. Discussion

Scarcity, low malignant potential, and indolent behavior are the main characteristics of SPN as previous studies mentioned. Lack of known risk factors for malignancy can lead to favorable prognosis and long survival rate of SPN [3, 11]. Nevertheless, the incidence of metastatic lesions and recurrence is 10-15% [3, 4]. A high ki67 index may exhibit the probability of malignant course and metastases [2, 6, 12]. SPN can affect predominantly young females in their second to fourth decade [6, 11]. Some authors believed that sex hormones might play a role in the development of the disease due to the notable presence of progesterone receptors [3, 6, 12]. Patients usually present with nonspecific clinical manifestations related to intra-abdominal mass such as pain, early fullness, dyspepsia, nausea, and vomiting; although most patients are asymptomatic, they are diagnosed incidentally on examination or abdominal imaging. However, in our case, the sharp and itchy acute abdomen forced the patient to refer to the emergency ward a couple of times which distinguished our patient from other reported cases [2, 9, 12, 13]. Although obstructive jaundice is a common symptom in the head pancreatic tumor, its presence is so rare in SPN patients [14] that guides us to discriminate between SPN and pancreatic cancer [13]. The importance of precise diagnosis is apparent because of curative treatment. Pathologic examination and immunohistochemistry (IHC) are useful methods used for appropriate diagnosis. IHC can distinguish SPN from pancreatic neuroendocrine neoplasm with positive staining for B-catenin, vimentin, and progesterone [14]. Under a microscope, beside specific features, SPNs generally show a heterogeneous appearance, numerous monomorphic neoplastic cells surrounded by branching capillaries, noncohesive neoplastic cells (with pleomorphic nuclei), and nuclear grooving plus cercariform cells suggested a significant clue to distinguish SPN from Peripheral Primitive Neuroectodermal Tumor (PNET) [11]. In radiologic investigations, CT is a useful modality that reveals a well-defined heterogeneous hypoechoic lesion [15] with peripheral contrast enhancement of pseudocapsule [16]. Also, endoscopic ultrasound-guided is a well-known and approved diagnostic tool in the differentiation of pancreatic lesions such as ductal pancreatic adenocarcinoma (PDAC) from neuroendocrine pancreatic tumors and other rare pancreatic neoplasms especially SPNs [13, 14]. However, EUS-guided Fine Needle Aspiration (EUS-FNA) as a safe tool in SPN enhancing the preoperative diagnostic efficiency with a sensitivity of 90.1% and specificity of 100% was not indicated due to obvious surgical indication. In our patient, eventually, EUS suggested SPN's diagnosis. Surgical resection has continuously been the treatment of choice for SPN patients; however, the location, size, and the local invasion of the tumor determine the surgical technique [1]. Depending on the tumor's location, pancreaticoduodenectomy is indicated for a tumor in the head of the pancreas, distal pancreatectomy for tail lesions, and the tumors in the neck and body can be treated by central pancreatectomy [12]. Recently, laparoscopic procedures and the parenchyma-preserving surgical approach are discussed in many pieces of literature [12, 13, 17–19]. Hao et al. recommended minimally invasive surgery for SPN of the proximal pancreas due to some convinced aspects like shorter hospitalization, decreased blood loss, and less transfusion requirement than open surgery [10, 17]. Likewise, other authors confirmed the success, safety, and feasibility of laparoscopic procedures in their patients [20]. Nevertheless, within the oncologic parameters into negative surgical margins, both approaches yielded similar long-term outcomes [10, 17].

Although many researchers demonstrated the superiority of laparoscopic technique as long as an experienced surgeon performed in distal lesions [10], it is not determined in the pancreas' proximal and central tumors [21]. Lin et al. demonstrated that the parenchyma-preserving surgical approach might be more appropriate for small SPNs of head pancreatic; however, another study believes that tumor recurrence is more likely in this procedure [13, 19]. Moreover, postoperative fistula complications occur more in patients who underwent parenchyma-preserving surgery, which did not occur in open surgery [13]. On the other hand, the results of a systematic review showed the absence of any significant differences in postoperative morbidity and postoperative fistula rates between open and minimally invasive pancreatectomy [10]. The concomitant incidence of SPNs with splenic cyst is not frequently reported, and no associations have been found between them as Hajjar et al. mentioned [2].

## 4. Conclusion

Solid pseudopapillary neoplasms of the pancreas are known as extremely rare entities with a predilection for young women. Surgical resection is the only curative treatment with an excellent long-term prognosis. Minimally invasive pancreatectomy could be a more effective option than open surgery.

## Data Availability

The supporting data for our findings are available by contacting the corresponding author.
